# Semantic Interoperability between IEC 61850 and oneM2M for IoT-Enabled Smart Grids

**DOI:** 10.3390/s21072571

**Published:** 2021-04-06

**Authors:** Salvatore Cavalieri

**Affiliations:** Department of Electrical Electronic and Computer Engineering, University of Catania, Viale A. Doria 6, 95125 Catania, Italy; salvatore.cavalieri@unict.it

**Keywords:** smart grid, internet of things, IEC 61850, oneM2M, semantic interoperability, SAREF

## Abstract

In the era of Industry 4.0, pervasive adoption of communication technologies based on the Internet of Things represents a very strong requirement in several domains. In the smart grid domain, there is the need to overcome one of the main limitations of the current electric grid, allowing the use of heterogeneous devices capable of measuring, monitoring and exchanging information about grid components. For this reason, current literature often presents research activities about enabling internet of things (IoT) in smart grids; in particular, several proposals aim to realize interworking between IoT and smart grid communication standards, allowing exchange of information between IoT devices and the electrical grid components. Semantic interoperability should be achieved in an interworking solution in order to provide a common meaning of the data exchanged by heterogeneous devices, even if they belong to different domains. Until now, semantic interoperability remains an open challenge in the smart grid field. The paper aims to propose a novel solution of interworking between two of the most used communication systems in smart grids and IoT domains, i.e., IEC 61850 and oneM2M, respectively. A semantic interoperability solution is also proposed to be used in the interworking scheme here presented.

## 1. Introduction

The smart grid (SG) concept is based on a new vision of the electric grid, which includes the maximization of the distribution of energy demand, the minimization of losses and the integration of renewable energy sources on a large scale, as pointed out in [1,2,3]. SG aims to overcome one of the main limitations of the current electric grid, related to the lack of integration of novel information and communication technologies, allowing the use of heterogeneous devices capable of measuring, monitoring and exchanging information about grid components, in order to make it more connected and smarter. Among the novel information and communication technologies there are those based on the internet of things (IoT), which play a strategic role in the era of Industry 4.0 as shown by [4,5,6,7,8]. The current literature presents several studies about enabling enabling internet of things (IoT) in SG, typically referred as IoT-enabled SG [9,10]. Several studies highlight the benefits that the IoT would bring within a smart grid; the reader may refer to the information given in [10,11,12,13,14,15] in order to have an overview of the advantages of introducing IoT technologies into a SG.

Among the main requirements in the IoT-enabled SG, there is the interworking between IoT and SG communication standards. Internetworking is the process of connecting different networks by using intermediary devices such as routers or gateways. Interworking between IoT and SG communication systems may allow IoT devices (e.g., controllers) to access information maintained by a server inside the SG domain and related to the status of the grid components.

Interoperability among heterogeneous devices is an imperative requirement for the realization of interworking solutions. In particular, semantic interoperability represents the highest level of interoperability, aiming to provide a common meaning of the data exchanged by heterogeneous devices, even if they belong to different domains [16]. Among the main initiatives to achieve semantic interoperability, there is that provided by the European Telecommunications Standard Institute (ETSI). It is currently focused on a standardization activity aimed to the definition of an open ontology called Smart Appliances REFerence (SAREF) to enable information sharing and semantic data understanding among IoT devices and servers using different technologies among several domains [17]. The scope of SAREF starts from smart appliances and extends to other domains like energy domain; SAREF4ENER is the extension of SAREF ontology in the energy domain [18]. In [19], SAREF/SAREF4ENER is proposed as ontology to be adopted into the SG in order to achieve the semantic interoperability. Although other studies aimed to realize semantic interoperability in IoT-enabled SG are present in the current literature, semantic interoperability remains an open challenge in this field, as pointed out in [20,21].

The paper aims to propose a novel interoperable interworking scheme in a IoT-enabled SG. Interworking of two of the most known and widely used communication standards in SG and IoT are considered, enriching this interworking by a semantic interoperability solution. In particular, the international standard IEC 61850 is considered in the SG domain, as it is recognized as a globally accepted solution in the power system automation domain [22]. In IoT domain, oneM2M communication standard is considered, as it represents one of the main initiatives conceived for solving the fragmentation of IoT landscape [23,24].

Integration of IEC 61850 and onM2M is proposed in this paper through the definition of an interworking architecture based on the standard oneM2M interworking proxy application entity (IPE) [25,26]. The proposed solution aims to allow IoT devices and applications in the oneM2M domain to access data and to invoke services available in the IEC 61850 domain. The interworking solution proposed between IEC 61850 and oneM2M has been enriched by a semantic interoperability approach, as said before. It is based on the definition of a common ontology, able to put into relationship the elements belonging to the ontologies adopted by the two interworked communication systems; this allows an exchange of information between the two different domains, allowing devices of the domains to understand the meaning of the data exchanged. The oneM2M standard already features an ontology called oneM2M base ontology [27]. Due to the lack of an ontology associated to IEC 61850 standard, an ontology has been ad-hoc defined for this communication standard, as an extension of SAREF4ENER ontology; the novel ontology, called IEC61850-SAREF4ENER, is presented in this paper. A common ontology between IEC61850-SAREF4ENER and the oneM2M base ontology has been defined and is presented in the paper; according to the semantic interoperability approach proposed, this common ontology is adopted for the interworking solution between IEC 61850 and oneM2M.

From the engineering design point of view, the paper aims to define an interworking proxy able to map the digital representation of each IEC 61850 electrical resource into another digital representation compliant with oneM2M standard. This design allows a generic IoT device and/or application based on oneM2M standard to access information maintained by IEC 61850 Server and related to the smart grid devices. The architecture of the proxy will be clearly described in the paper; emphasis on the semantic interoperability of the interworking scheme realized by the proxy will be given.

The proposal presented in the paper has been defined following a methodological approach based on the steps here described. A state-of-the-art has been realized at the beginning; it focused on the analysis of existing interworking solutions between IEC 61850 and IoT communication systems. The analysis pointed out that interworking solutions involving IEC 61850 exist but none of them was also relevant to oneM2M; in any case the existing solutions did not take into account semantic interoperability. This analysis was very important has it allowed to justify the need for the research carried out; it will be deepened in Section 2. In order to be able to propose an interoperable interworking solution between IEC 61850 and oneM2M, the standard oneM2M-based interworking schema has been adopted in the research; alternative possible choices to adopt proprietary solutions were avoided. As the oneM2M-based Interworking schema requires the presence of ontologies for the two interworking domains, definition of an ontology for the IEC 61850 was necessary due to the relevant lack. An ontology called IEC61850-SAREF4ENER was defined for the IEC 61850; Section 4 is aimed to the description of this ontology (after a brief description of the IEC 61850 standard given in Section 3). Another requirement of the oneM2M-based interworking schema here adopted was the definition of a common ontology between the ontologies for the two interworking domains. For this reason, the research activity here described, defined this common ontology which will be described by Section 6. The next and last step achieved in the methodological approach here presented was the definition of the interoperable interworking solution, which will be described by Section 7.

## 2. Related Work

As pointed out in the Introduction, the proposal is aimed to present a method able to realize two goals. The first is the definition of an interworking scheme between IEC 61850 and oneM2M; the other goal is the definition of a solution to enrich this interworking with semantic interoperability. The aim of this section is to compare the proposed method with other approaches present in literature. In short, it will be shown that interworking between IEC 61850 and IoT communication systems exists in the current literature, but none of them considers semantic interoperability. Moreover, this section will point out that interworking between IEC 61850 and oneM2M is original, as no other solutions exist about this issue. Finally, this section will highlight that the semantic interoperability solution here proposed and based on the use of the oneM2M base ontology it is adopted in different domains, but none of them are relevant to SG.

The literature presents several approaches aimed to enable interworking between IoT and SG domains, but the most part of them does not consider semantic interoperability. In [28] a mapping between CoAP and IEC 61850-based substation automation systems in a SG environment is proposed. In [29] an IEC 61850 and XMPP communication-based energy management in microgrids with integrated electric vehicles is introduced. Mapping the IEC 61850 to RESTful Services is presented in [30]. In [31] the authors present a review of the IoT protocols used in the SG aiming to achieve guidelines in utilizing proper IoT protocol that can meet the SG requirements; addressing effective elements in applying IoT in the SG’s future trends is another contribution of this paper. In [32] authors discuss integration of IEC 61850 and IoT, without considering semantic aspects. In [33] the authors highlight that a semantic description of data is strongly needed (but not currently available) in present different scenarios, including SG, where heterogeneous applications need to exchange huge amount of data without knowing how they are represented. In [34] the semantic interoperability is considered for a Microgrid control system scenario, but not specific solutions for the IoT and SG interworking are presented.

Introduction pointed out that the semantic interoperability here proposed is based on the use of the oneM2M base ontology. Literature presents several papers dealing with oneM2M ontology and its use for semantic interoperability between different domains, but none of them are relevant to SG. In [35] authors propose an oneM2M interworking proxy entity for ECHONET lite protocol able to allow interworking between devices from different domains via the oneM2M ecosystem; adoption of oneM2M ontology for the ECHONET Lite resources is also proposed. In [36] authors demonstrate how ontology-based device descriptions can improve the interoperability in the IoT through oneM2M ontology. In [37] the extension of the oneM2M standard to support semantic data interoperability based on IoT-O is discussed and the authors propose its validation in SG as a future work.

## 3. The IEC 61850 Standard

IEC 61850 is the leading standard for substation automation; more information about this standard can be achieved by [22,38,39]. It is intended to structure the intelligence of protection, control, monitoring and automation functions. The standard fulfils automation architecture requirements for utility subsystems, enabling communication among multi-vendor equipment. The following subsections describe the main features.

### 3.1. IEC 61850 Information Model

IEC 61850 defines an object-oriented modelling of process data required for the power system automation. IEC 61850 information model has been primarily defined for the exchange of information within substations by the document IEC 61850-7-4 [38]. IEC 61850 information model is now extended for other domains, among which one can include distributed energy resources (DER)s by IEC 61850-7-420 [38].

**Figure 1:**
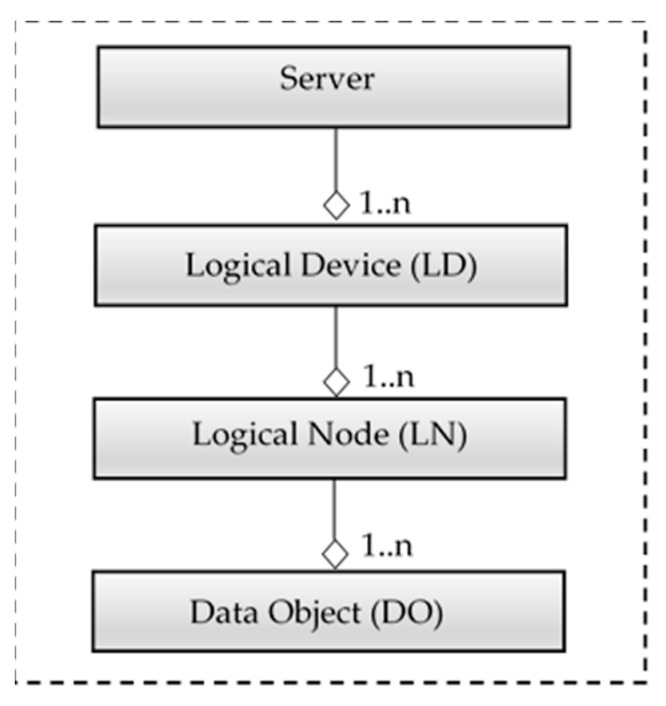
IEC 61850 Information Model.

Figure 1 shows the main elements of the IEC 61850 information model. The top parent class is the server, which is hosted by physical device, i.e., the controller part of the device, which is also called the intelligent electronic device (IED).

The server consists of one or more logical devices (LDs), i.e., virtual representations of devices intended for supervision, protection or control of automated systems. LDs are created by combining several logical nodes (LNs) which represent various device functionality interfaces. Data objects (DOs) are representations of the common information of logical nodes. DOs are typed by common data classes (CDC) from IEC 61850-7-3 or IEC 61850-7-2 [38].

Each CDC describes the type and structure of the data within the LN. For instance, there are CDCs for status information, measured information, controllable status information, controllable set point information and different kinds of settings. Each CDC has a defined name (CDC-ID) and a set of CDC attributes (DataAttributes); each individual attribute belongs to a set of functional constraints (FC) that groups the attributes into categories. Finally, each individual attribute of a CDC may be mandatory, optional, or conditional; this is expressed by a particular value of a field called m/o/c. For example, the common data class single-point status (SPS) defined by IEC 61850-7-3 [38], features several data attributes, among which there are: stVal (status value), q (quality), and t (time stamp) with the functional constraint ST (status information); they are mandatory (m/o/c = M).

In IEC 61850-7-2 [38], a generic Class, GenClass, is defined, which abstracts the IEC 61850 information model. Table 1 summarizes the key concepts of the GenClass for each element of the information model, pointing out the main attributes and services.

A logical device is abstracted as GenLogicalDeviceClass. It consists of LDName which unambiguously defines the name of an instance of a logical device. LogicalNode [1...n] is a list of n logical nodes contained in the logical device. Services which may be applied for this class include the GetLogicalDeviceDirectory service to retrieve references (called ObjectReferences) of all LNs belonging to the LD.

The generic representation of a Logical Node is done via GenLogicalNodeClass, which consists of a unique name, LNName, LNRef representing the path of that LN (according to the syntax defined for the CommonACSIType in IEC 61850-7-4 [38]), and DataObject contained by the instance of that LN. The services allowed for GenLogicalNodeClass are GetLogicalNodeDirectory, that is able to retrieve the ObjectReferences contained in the LN instance, and GetAllDataValues, which returns the values of the attributes that have a certain functional constraint (FC).

The GenDataObjectClass represents a generic DataObject defined via CDC. It consists of DataObjectName, DataObjectRef, an enumerative attribute m/o/c (featuring the values mandatory, optional, conditional), and DataObjectType which is an instance of GenCommonDataClass type. Several services are defined for this class, as shown by Table 1.

GenCommonDataClass represents a CDC, which features a defined name (CDC-ID) and a set of optional CDC attributes (DataAttributes), as said before.

GenDataAttributeClass is an abstract representation of a data attribute; a data attribute consists of a name (i.e., DataAttributeName), a functional constraint (FC) specifying use of data attribute and a property called m/o/c.

### 3.2. IEC 61850 SCL

The IEC 61850 standard provides a data exchange mechanism using an XML-based file format called Substation Configuration Language (SCL). SCL allows the representation of an IEC 61850 system configuration of electrical substation devices, including representation of data and communication services, as described in [38,39]. Its main purpose is to allow an interoperable exchange of communication system configuration data between an IED configuration tool and a system configuration tool from different manufacturers. The SCL file is divided into sections, each of which provides different information. Header section includes general topics and details about SCL and file version. Communication section contains communication details. Substation section describes electrical topology for substation or any other electrical process. IED section describes devices capacities, functions and data it manages. DataTypeTemplates section describes functional data model by defining LNs, data objects and data attributes.

## 4. Ontology Definition for IEC 61850

Interoperability based on the definition of a common ontology is a very strong requirement in the field of smart grid. Literature addresses several papers about this issue.

In [19] authors have investigated the need for alignment among the communication standards in smart grid (focusing on demand side flexibility, DSF) from the utility, telecommunication and home appliances industries; among the standards considered there are: CEN 16836 (ZigBee SEP2), CENELEC EN 50491-11 Smart Metering, CENELEC EN 50631-1 (SPINE), IEC 61968-9 CIM for metering, IEC 61970 CIM, IEC/CENELEC 62056 COSEM, CENELEC EN 50090 (KNX). They concluded that alignment between DSF standards is strongly required; furthermore, their analysis pointed out that the ontology defined by SAREF [17] and its extension in the SG, called SAREF4ENER [18], could be used as the overarching ontology to facilitate this alignment.

In [40], the SARGON—SmArt eneRGy dOmain oNtology is presented; it extends SAREF to cross-cut domain-specific information representing the smart energy domain and includes building and electrical grid automation together.

To the best of the author’s knowledge, current literature does not present any proposal about alignment between IEC 61850 and SAREF/SAREF4ENER ontology.

On the basis of what written until now, the author proposes the alignment of SAREF ontology with IEC 61850 standard, extending the SARE4ENER ontology presented in [18,41] in order to include the IEC 61850 concepts. This section aims to describe this extension; in the following it will be simple called “IEC61850-SAREF4ENER”. Before the description of the proposal, a brief overview on SAREF and SAREF4ENER ontology will be given.

### 4.1. SAREF and SAREF4ENER Ontology

The European Telecommunications Standards Institute (ETSI) is currently focusing on the definition of an open ontology to enable information sharing and semantic data understanding among IoT devices and servers; this ontology is called Smart Applications REFerence (SAREF) [17,42]. In order to enable interoperability among several domains, extensions of SAREF have been defined. In the energy domain, SAREF4ENER has been defined in collaboration with Energy@Home (http://www.energy-home.it (accessed on 27 March 2021)) and EEBus (http://www.eebus.org/en (accessed on 27 March 2021)), the major Italy- and Germany-based industry associations, to enable the interconnection of their (different) data models [18,41].

SAREF focuses on the concept of device, which is defined as “a tangible object designed to accomplish a particular task in households, common public buildings or offices. In order to accomplish this task, the device performs one or more functions” [42]. Examples of devices are light switch, temperature sensor, energy meter and washing machine. The saref:Device class is defined according to this definition. Among the devices defined by SAREF, there is that describing a washing machine (saref:WashingMachine class); it is designed to wash (task) and to accomplish this task it performs a start and stop function [42].

A device may consist of other devices; this is represented by the saref:consistsOf relationships between the devices.

A saref:Device may be featured by properties whose values may be achieved by real measurement. The classes saref:Measurement and saref:Property allow to relate different measurements from a given device for different properties. In particular, the saref:Measurement class describes a measurement of a physical quantity for a given saref:Property. In this way, it is possible to differentiate between properties and the measurements made for such properties. The saref:MeasuresProperty relationship allow to link a saref:Device class to a relevant saref:Property class, which in turn may relate to a saref:Measurement, through the saref:relatesToMeasurement relationship.

A device could offer services, each of which is a representation of a function to a network that makes the function discoverable, registerable, and remotely controllable by other devices in the network. A service is represented in SAREF with saref:Service class; the relationship between the saref:Device and the relevant saref:Service class is given by the saref:offers relation. The relationship between the saref:Service and the relevant saref:Function is the saref:represents relation. A function is represented in SAREF with the saref:Function class and it is defined as the functionality necessary to accomplish the task for which a Device is designed. Examples of functions are the saref:ActuatingFunction and saref:MeteringFunction, which will be used in the proposal here presented. A saref:Function shall have at least one command associated to it, represented by saref:Command class; the relationship between the saref:Function class and the relevant saref:Command class is given by the saref:hasCommand relation. Among the available commands there is the saref:GetCommand class which only gives a directive to retrieve a certain value.

In SAREF4ENER ontology several subclasses have been created starting from the SAREF classes, in order to meet the specific requirements in the smart grid domain [41]. The SAREF4ENER ontology starts from the saref:Device class concept. A s4ener:device is a subclass of a saref:Device, i.e., it inherits the properties of the more general device defined by SAREF and extends it with additional properties that are specific for SAREF4ENER.

### 4.2. Extending SAREF4ENER Ontology for IEC 61850

**Figure 2:**
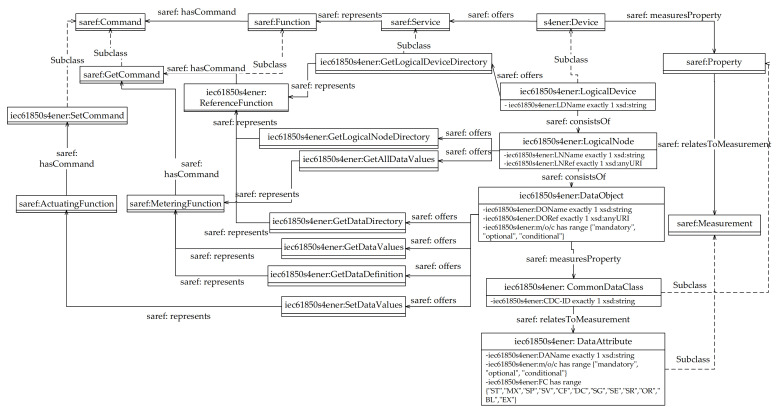
IEC61950-SAREF4ENER Ontology.

Figure 2 shows the elements introduced in the IEC61850-SAREF4ENER ontology here proposed; their names feature the prefix “iec61850s4ener”, in order to be differentiated by those defined in SAREF (using the prefix “saref”) and in SAREF4ENER (using the prefix “s4ener”).

An iec61850s4ener:LogicalDevice class has been defined in IEC61850-SAREF4ENER ontology to represent the IEC 61850 GenLogicalDevice Class (described by Table 1); it is a subclass of s4ener:Device. An iec61850s4ener:LogicalDevice has exactly one data property represented by iec61850s4ener:LDName defined as xsd:string. As the IEC 61850 GenLogicalDevice Class is featured by one or more LogicalNode elements, the class iec61850s4ener:LogicalNode has been defined as a subclass of iec61850s4ener:LogicalDevice. In order to represent the relationship between IEC 61850 GenLogicalDevice Class and the LogicalNode elements, Figure 2 shows the original saref:consistsOf (described in [42]) used between the two above mentioned iec61850s4ener classes.

As showed by Table 1, the IEC 61850 GetLogicalDeviceDirectory service is defined for the logical device; this service is represented by the iec61850s4ener:GetLogicalDeviceDirectory class which is a subclass of saref:Service. According to the IEC 61850 standard, the aim of this service is that to retrieve all LN references of a logical device; for this reason, the iec61850s4ener:ReferenceFunction class has been introduced in the proposed ontology as a subclass of saref:Function. This function has a specific command represented by saref:GetCommand in order to retrieve the requested references.

As said before, the iec61850s4ener:LogicalNode class has been defined in the proposed ontology as a subclass of iec61850s4ener:LogicalDevice, in order to represent IEC 61840 Logical Nodes contained in a Logical Device. This iec61850s4ener class has exactly two data properties, i.e., LNName (defined has xsd:string) and a LNRef (defined as xsd:anyURI). According to Table 1, an iec61850s4ener:LogicalNode class features two services: iec61850s4ener:GetLogicalNodeDirectory and iec61850s4ener:GetAllDataValues; they have been defined as Subclass of saref:Service (not shown in Figure 2 for space reason). The first one represents the IEC 61850 GetLogicalNodeDirectory, which has the aim to retrieve all DataObject references; for this reason, the iec61850s4ener:GetLogicalNodeDirectory is the representation of the saref:ReferenceFunction class with saref:GetCommand command, as shown by Figure 2.

The iec61850s4ener:GetAllDataValues represents the IEC GetAllDataValues, which returns the values of attributes; for this reason, iec61850s4ener:GetAllDataValues is the representation of the saref:MeteringFunction (in order to measure values) with saref:GetCommand command (in order to get values).

As the IEC 61850 logical node is made up by several DataObject, the iec61850s4ener:DataObject class has been defined to represent the IEC 61850 DataObject. It has three data properties: a unique DOName represented as xsd:string, an DORef represented as xsd:anyUri and an iec61850s4ener:m/o/c represented as enumeration type (with three possible values: “mandatory”, “optional” and “conditional”).

As said in Section 3.1, CDC is the common data class that defines the structure of the IEC 61850 DataObject. For this reason, the novel class named iec61850s4ener:CommonDataClass has been introduced into the extended ontology presented in this paper; it has been defined as a subclass of saref:Property. The iec61850s4ener:CommonDataClass has exactly one data property named CDC-ID (represented as xsd:string). Recalling that a CDC is a set of IEC 61850 DataAttributes, the iec61850s4ener:DataAttribute class has been defined as subclass of saref:Measurement and it consists of three data properties: DAName (defined as xsd:string), m/o/c (defined as enumeration type, with the values given by Figure 2) and FC (defined as enumeration type, with the values given by the same figure). These data properties model the relevant attributes of the IEC 61850 DataAttributes.

Services of iec61850s4ener:DataObject are iec61850s4ener:GetDataValues, iec61850s4ener:SetDataValues, iec61850s4ener:GetDataDefinition and iec61850s4ener: GetDataDirectory, defined as subclasses of saref:Service. The iec61850s4ener:GetDataValues and iec61850s4ener:GetDataDefinition are representation of saref:MeteringFunction. The iec61850s4ener:SetDataValues service is representation of the saref:ActuatingFunction able to transmit data by the newly defined iec61850s4ener:SetCommand class command. The iec61850s4ener:GetDataDirectory service is representation of the iec61850s4ener:ReferenceFunction, as shown by Figure 2.

The extended ontology here presented has been defined through Protegè tool [43]. In the GitHub repository whose address is given by [44], the extended ontology is freely available.

## 5. The oneM2M Standard

**Figure 3:**
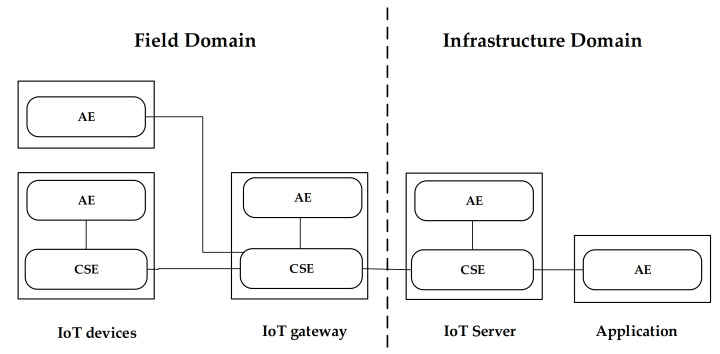
oneM2M Infrastructure and Field Domains.

The oneM2M communication system provides interoperability support for IoT technologies, through a reference architecture model, according to which the IoT environment is divided into two domains: infrastructure and field. Infrastructure domain is the domain in which servers and applications (e.g., control, monitoring) reside. Field domain contains the oneM2M-compliant IoT devices exchanging data with the servers and applications located at the infrastructure domain; communication with the infrastructure domain may be realized also through one or more IoT gateways located in the field domain. Figure 3 shows these domains.

The oneM2M reference architecture model is made up by three layers: application, common service and network service [45]. Each layer is represented as an entity in the oneM2M system. Application entity (AE) represents application services located in a IoT device, IoT gateway, server or application; it is defined as an entity in the application layer implementing an application service logic. Common service entity (CSE) represents an instantiation of a set of common service functions with which the oneM2M platform provides common services for the IoT service environments; an example is represented by oneM2M data management services. CSE may be located in a IoT device, IoT gateway and server, as shown by Figure 3.

The oneM2M architecture adopts a resource-oriented architecture (ROA) model, and thus the services and data that oneM2M system supports are managed and exposed as a resource information model, as described in [45,46]. The oneM2M resources are specified in association with a CSE; the resources are the representation in the CSE of the components and elements within the oneM2M system. According to the ROA concept, resources can be uniquely addressed by the uniform resource identifier (URI) and the interaction with the resources are supported by the basic four CRUD (create, retrieve, update, and delete) operations. According to the current version of the oneM2M specifications, CRUD operations may be realized for example by HTTP methods [47]. Each oneM2M resource is made up by set of mandatory and optional attributes, whose description may be found in [45,46].

**Figure 4:**
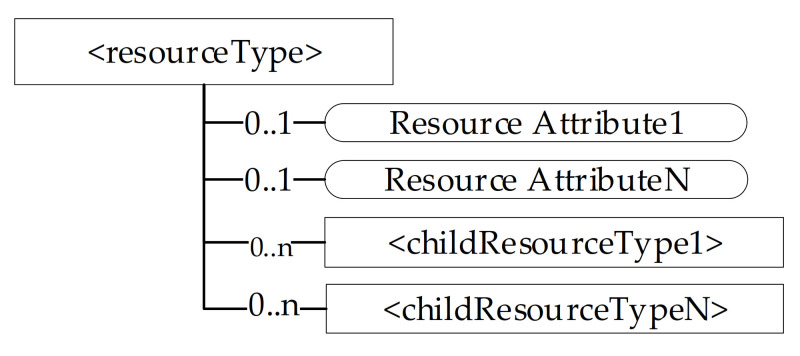
Hierarchical structure of a oneM2M resource.

The oneM2M system manages its resources through a hierarchical structure; Figure 4 points out that each resource of a certain type features a hierarchical structure made up by attributes and child resources.

**Figure 5:**
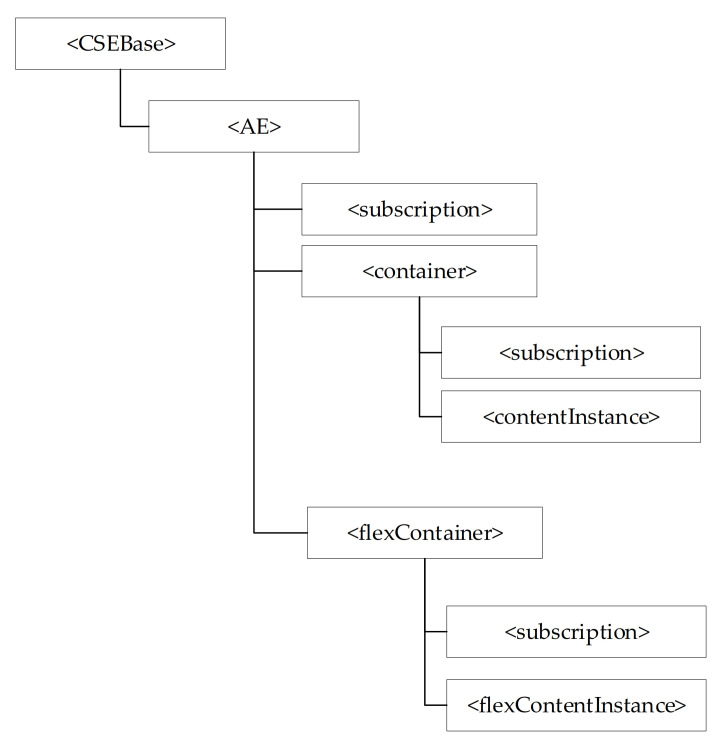
Common Service Entity (CSE) resource tree.

Considering the CSE hosting oneM2M resources, these are organised through a resource tree whose root is realized by a particular resource of type <CSEBase>. Resources are created as child of resources, which represent service and data in the oneM2M system. Figure 5 shows an example of the hierarchical structure of resources inside oneM2M system as it can be seen it is a resource tree having a resource of <CSEBase> type as root. In the following, the resource types defined in oneM2M standard will be described, giving more emphasis to those used in the paper.

A <CSEBase> resource represents a CSE. As said before, a resource of <CSEBase> type is the root of the resource tree which organises the resources of a oneM2M system inside a CSE.

In order an AE could access the oneM2M resources maintained inside a CSE, it is necessary that it must be registered in the CSE hosting the oneM2M resource tree. This is realized by the creation of a <AE> resource inside the CSE resource tree. According to [45] a registrar CSE is the CSE where an AE has been registered. An <AE> resource represents information about an application entity registered to a CSE.

The <container> resource represents a container for data instances; it is used to share information with other entities and potentially to track the data. The <contentInstance> resource represents a data instance in the <container> resource.

The <flexContainer> resource type is a customizable container for data instances; like <container> it is used to share information with other entities and potentially to track the data. A <flexContainer> resource could have customAttributes; the attribute name and attribute data type of customAttributes are ad-hoc defined for each specialization of <flexContainer>.

The concept of subscription to resource instances is also specified in oneM2M. A subscription is used in order to receive notifications about content changes; it allows efficient monitoring of resource instances and thus of the exposed resources. In particular, the resource defined as <subscription> contains subscription information. It is represented as a child-resource of the subscribed-to resource, and it contains information about the subscriber and notification policies [45]. Create <subscription> request service is used to create such resource, as described in [46].

**Figure 6:**
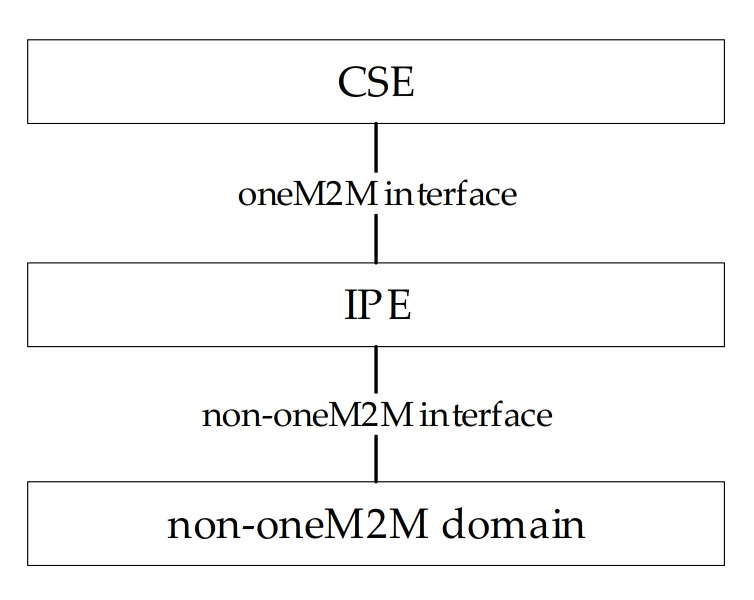
Interworking Proxy application Entity (IPE).

In order to enhance interworking, oneM2M uses a specialized interworking application entity called interworking proxy application entity (IPE). An IPE is an AE that supports both oneM2M and the non-oneM2M interfaces towards oneM2M and non-oneM2M domains, as shown by Figure 6; more information about IPE may be achieved in [48]. The IPE is characterized by the capability of remapping the related non-oneM2M information model to the oneM2M resources. Depending on the complexity of the non-oneM2M information model, this can imply that the IPE constructs a complex set of resources (built from the basic oneM2M resources) in the CSE. These resources are oneM2M representations of the non-oneM2M information model and they enable CSEs and AEs to access the entities in the non-oneM2M via the IPE.

### 5.1. The oneM2M Base Ontology

The oneM2M standard includes an ontology called “oneM2M Base Ontology “, defined by [49] and available in [50] in descriptive format (OWL). The oneM2M base ontology defines entities and relationships; an entity is defined by a Class, whilst a relationship is realized by a property. Among the available properties there is the “Object Property” which allows to realize a relationship between classes.

Classes may have subclasses that represent more specific concepts than the superclass; in oneM2M base ontology a particular relationship between two classes named “is-a” indicates an inheritance of the classes.

**Figure 7:**
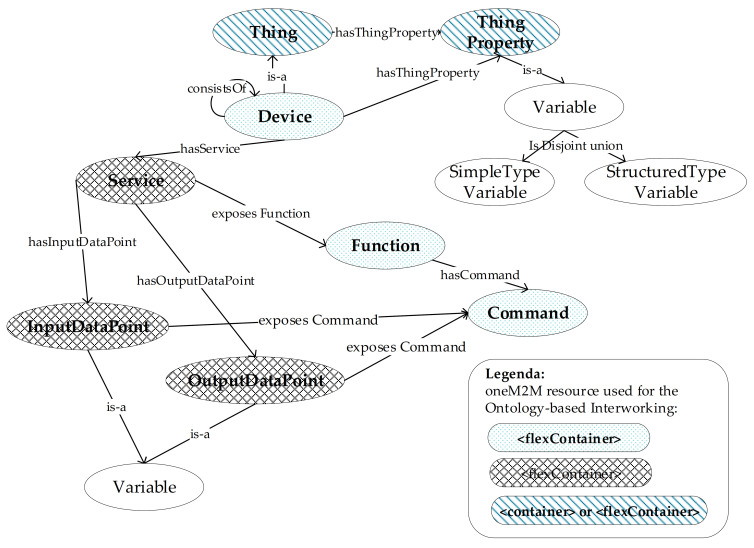
Classes and Properties of oneM2M Base Ontology.

Figure 7 shows some of the available classes and properties in the oneM2M Base Ontology. The nodes (ellipses) denote classes whereas edges (arrows) denote properties; Figure 7 points out only the classes and properties used in the paper; for more details the reader may refer to [49,51].

The thing class is an entity that can be identified in the oneM2M System. A Thing may have properties, represented by the ThingProperty Class; in this case the hasThingProperty Object Property is used to put into relationship thing and ThingProperty classes. For example, a room modelled in oneM2M would be a Thing that could have a room-temperature as a ThingProperty (via hasThingProperty).

A ThingProperty Class features values, e.g., the indoor temperature of the room could be a value of a thing “room”. ThingProperty’s values belongs to a variable class (shown in Figure 7), which may feature several sub-classes; among available sub-classes, Figure 7 shows the SimpleTypeVariable Class that consists of variables belonging to simple xml types like xsd:integer and xsd:string. StructuredTypeVariable Class is another sub-class of Variable Class (shown in Figure 7) that describes a structured variable made up by a set of other variables.

In general, it is assumed that a thing is not able to communicate electronically with its environment. The sub-class of thing that is able to perform this task is called a device class. A device can be composed of several (sub-)devices; this is represented by the consistsOf Object Property, shown in Figure 7. In particular, the consistsOf relationship shown by Figure 7 allows to define a composite entity that consists of other entities (e.g., a temperature/humidity sensor that consists of a temperature sensor and a humidity sensor).

A device contains a logic and is producer and/or consumer of data that are exchanged via its services with other entities in the network. A service is represented by service class and the hasService Object Property allows to represent the services of a device, as shown by Figure 7.

A Service if featured by InputDataPoint and OutputDataPoint Classes. OutputDataPoint Class is a variable that is set by a device in its environment and that provides state information about the service. The device updates the OutputDataPoint Variable autonomously (e.g., at periodic times). A third party may trigger the device to update the data of the OutputDataPoint; in any case the third part may retrieve the current value of a OutputDataPoint. An InputDataPoint Class is a variable that the device reads out autonomously. It is possible to enable a third party to instruct the device to retrieve (out of schedule) the current value of a InputDataPoint variable.

In order to represent tasks performed by a device, the function class has been defined. A command class represents an action that can be performed to support a particular function. The hasCommand Object Property specifies a command for each function.

A Function is exposed in the network as services of the device, making the function discoverable, registerable, remotely controllable in the network; a service can expose one or more functions. Service and function classes are linked by the “exposes Function” Object Property, as shown by Figure 7.

While a function describes the meaning of the device’s function, the service is used to describe how such a function is represented in a communication network and is therefore dependent on the technology of the network. For example, a function “turn_light_On_or_Off” could be exposed in the network by a service “Binary Value Actuator”.

### 5.2. The oneM2M Ontology-Based Interworking

This section pointed out that oneM2M standard uses a specialised application entity called IPE, in order to realize interworking between oneM2M and non-oneM2M domains. The oneM2M standard enables the semantic interoperability of this interworking solution, by the so-called oneM2M ontology-based Interworking described in [52,53,54,55]; the IPE is the main actor in the oneM2M semantic interoperability implementation, as it will be explained in the following.

One of the key concepts of oneM2M ontology-based interworking is that semantic interoperability can be enabled only if a mapping between the oneM2M base ontology and the non-oneM2M ontology is realized. OneM2M defines this mapping as “common ontology”. According to [54], common ontology consists in an OWL or RDF file where all the relationships between classes of the oneM2M base ontology and non-oneM2M ontology are defined. According to the common ontology each class of the non-oneM2M ontology corresponds to a class in the oneM2M base ontology.

Another key concept of the oneM2M ontology-based Interworking is that for each class of the oneM2M base ontology, certain oneM2M resources are allowed to be used to represent information relevant to the specific ontological class; as said in the previous section, this representation occurs into a CSE resource tree. The legenda of Figure 7 points out the oneM2M resources to be used for the oneM2M ontology-based interworking; for each oneM2M base ontology class the relevant oneM2M resource is shown (highlighting the possible choices when available). For space reason, the legenda does not present information about variable; oneM2M variables and the relevant sub-classes (e.g., SimpleTypeVariable and StructuredTypeVariable) may be realized through the customAttributes of <flexContainer> resources (described before in this section).

The last key concept of the oneM2M ontology-based interworking is how each interworked resource belonging to the non-oneM2M domain is represented by oneM2M resources. The procedure adopted will be explained in the following. In few words, for each non-oneM2M resource the relevant class in the non-oneM2M ontology is considered. On the basis of the relationships fixed in the common ontology, the corresponding oneM2M base ontology class is found. The oneM2M resource associated to this oneM2M base ontology class is used to represent the original non-oneM2M resource, in the oneM2M domain. All these operations are performed by the IPE, which plays a strategic role in the oneM2M ontology-based interworking. In order to make this process smarter, oneM2M ontology-based Interworking uses suitable schema files written in XML schema definition (XSD); more details may be achieved in [54]. For each non-oneM2M class, a schema describes the correspondence with the relevant oneM2M resource which must be used to represent the original class. An XML schema definition (XSD) is an abstract representation of an oneM2M object’s characteristics. Its main components regard element, child-element, attributes, and data type declarations. Components described provide the definition of custom object and its specialization using a oneM2M resource.

**Figure 8:**
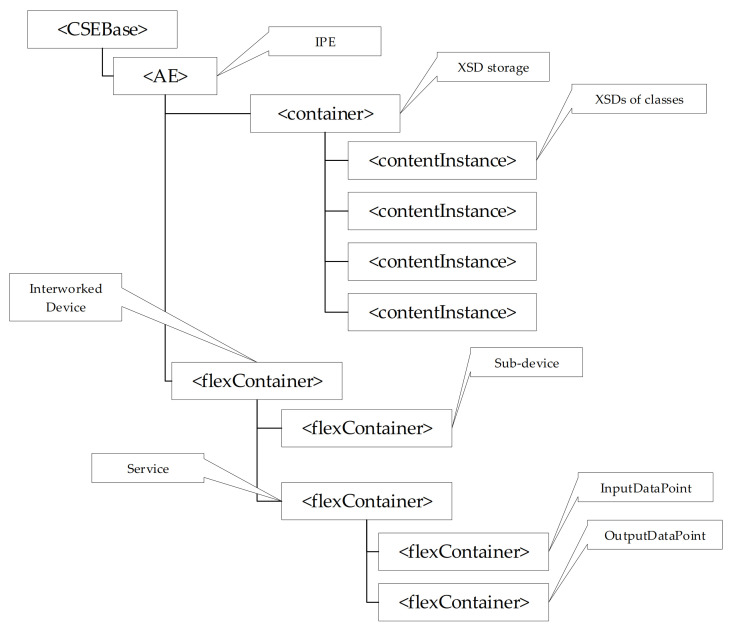
CSE structure in oneM2M-based Interworking.

In order the IPE may properly use a CSE to represent non-oneM2M resources, the oneM2M-based Interworking foresees a particular structure of the CSE. Figure 8 shows the CSE resource tree structure used to represent the non-oneM2M resources according to oneM2M-based interworking.

First of all, the IPE must be registered in the CSE; for this reason, an <AE> resource must be created as child of <CSEBase> resource to represent the IPE. Figure 8 points out the <CSEBase> resource related to the CSE hosting the oneM2M resources (representing those belonging to the non-oneM2M domain), and the <AE> resource representing the IPE. As said before, the XSD schemas are very important in order to allow the IPE to instantiate in the registrar CSE each oneM2M resource that will contain the elements mapping the non-oneM2M information model. The oneM2M-based interworking requires that all the schemas must be properly maintained in the CSE; in particular, a <container> child resource of the IPE <AE> resource must be created for holding XSD files (this is called the XSD storage, as shown by Figure 8). The XSD files are saved as <contentInstance> resources grouped inside the XSD storage. According to the legenda of Figure 7, an interworked device, belonging to the non-oneM2M domain, is represented by a <flexContainer> created as child- resource of <AE> of IPE. A <flexContainer> resource is also used for a sub-device (created as child-resource of the <flexContainer> of its parent device). Considering again the content of the legenda in Figure 7, <flexContainer> resources are used to represent Services and are created as child-resources of the <flexContainer> resource that represents the device or sub-device to which the Service belongs. InputDataPoint and OutputDataPoint are also modelled as <flexContainer> resources featuring customAttributes. Figure 8 shows the <flexContainer> resources just described.

In order to help the reader to better understand the oneM2M-based Interworking, a very simple example is presented. Let us assume to have a non-oneM2M domain where an ontology has been defined. In particular a washing machine device is considered for this non-oneM2M domain; it is assumed that it has been manufactured by the manufacturer ABC. The ontology that describes the ABC washing machine is identified by the IRI http://www.ABC.com/WashingMachine (accessed on 27 March 2021). It features the class ABC:ABC_WM representing the washing machine, and defined as a subclass of the saref:WashingMachine class, as described in [42]. Let us assume that a service exists for the Washing Machine defined by the class ABC:SwitchOnService which is a subclass of saref:SwitchOnService [42]. Finally it has been assumed that a description is given for the Washing Machine, defined by a class ABC:Description and represented as a string.

**Figure 9:**
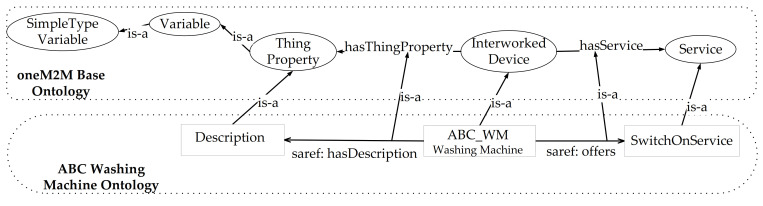
Common Ontology between oneM2M Base Ontology and SAREF-based Washing Machine Ontology.

It has been assumed that a common ontology is defined to map oneM2M Base Ontology and the SAREF-based ontology of the washing machine class. Figure 9 shows the common ontology, based on the mapping between classes and relationships of the two ontologies. As shown, the class ABC:ABC_WM is defined as subclass of oneM2M base ontology interworked device, the ABC:SwitchOnService is a subclass of oneM2M base ontology service, and ABC:Description is a subclass of oneM2M Base Ontology Thing Property. The same Figure 9 shows the relationships between saref:offers and oneM2M Base Ontology hasService Object Property, and between saref:hasDescription and oneM2M Base Ontology hasThingProperty Object Property.

**Figure 10:**
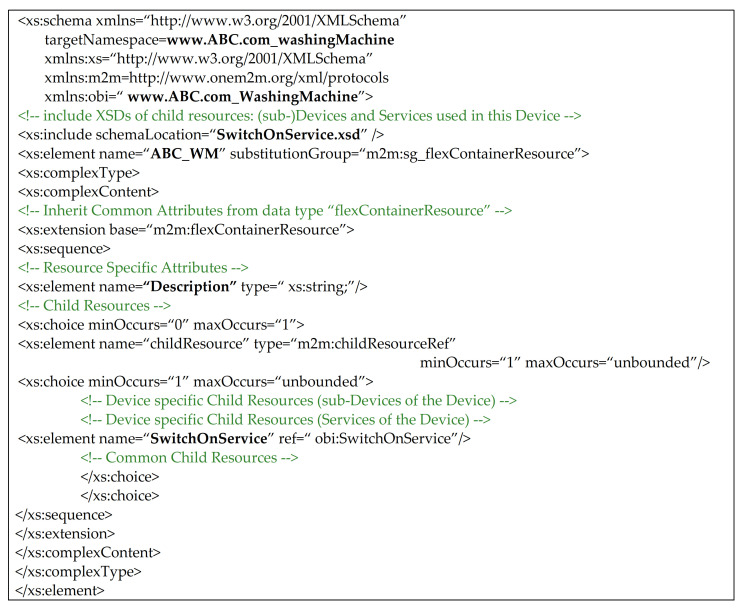
XSD file representing the ABC: ABC_WM class as a <flexContainer> oneM2M resource.

On the basis of this common ontology, XSD files relevant to the interworked device and the service may be created. For example, Figure 10 shows the XSD schema defined for the interworked device aimed to represent the ABC: ABC_WM class as a <flexContainer> oneM2M resource.

As it can be seen from Figure 10, the XSD schema is made up by several subsections. The first one contains the list of the XSD files describing the child oneM2M resources. On the basis of the common ontology given by Figure 9, it is clear that ABC:ABC_WM class is related to ABC:SwitchOnService. For this reason, the first section of XSD schema in Figure 10 must contains the XSD file related to the class saref:SwitchOnService, as shown by Figure 9. The second and last section of XSD schema shown in Figure 10, exactly defines the <flexContainer> structure that must be used to represent the ABC:ABC_WM class into oneM2M CSE. Furthermore this section describes the non-common attributes hold by this resource; there is only the Description attributed defined as xs:string, according to the common ontology shown by Figure 9. Then a particular subsection named “Child Resource” is present; it is aimed to define the schemas of the <flexContainer> child resources. In particular, it defines that this <flexContainer> features a sub-Device relevant to the Service SwitchOnService. Finally, child resources common to oneM2M are specified in the last part of this subsection; in this case it is empty.

**Figure 11:**
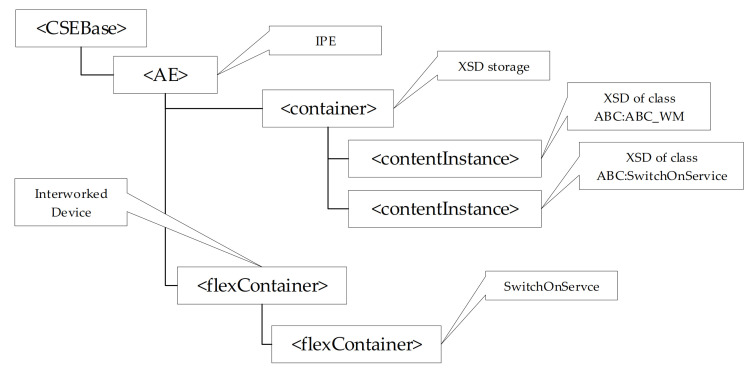
CSE structure considering the Common Ontology of Figure 9.

According to the common ontology shown by Figure 9 and on the XSD files relevant the ABC:ABC_WM and ABC:SwitchOnService, Figure 11 shows how the CSE could be filled by the IPE in order to represent in the oneM2M CSE, the interworked device and the relevant Service belonging to the ABX washing machine ontology.

## 6. The oneM2M and IEC61850-SAREF4ENER Common Ontology

In order to realize a semantic interoperability solution between IEC 61850 and oneM2M standards, the definition of a mapping between the relevant ontologies seems necessary. As said in Section 4, IEC 61850 does not feature an own ontology and for this reason the IEC61850-SEREF4ENER ontology has been defined in this paper. The aim of this section is that to present a common ontology between oneM2M base ontology and the extended IEC61850-SAREF4ENER ontology.

As said in the introduction, ETSI supports oneM2M global initiative and it is currently focused on standardization framework for SAREF ontology to enable information sharing and semantic data understanding among IoT devices. ETSI has already defined the mapping rule between SAREF and oneM2M base ontology in document [42], aligning the classes present in SAREF with those featuring the oneM2M base ontology. At the moment, this work doesn’t include SAREF4ENER concepts. Author have considered the rules already defined in document [42] and have been applied to the IEC61850-SAREF4ENER classes ad hoc defined for the IEC 61850 interworking scenario.

**Figure 12:**
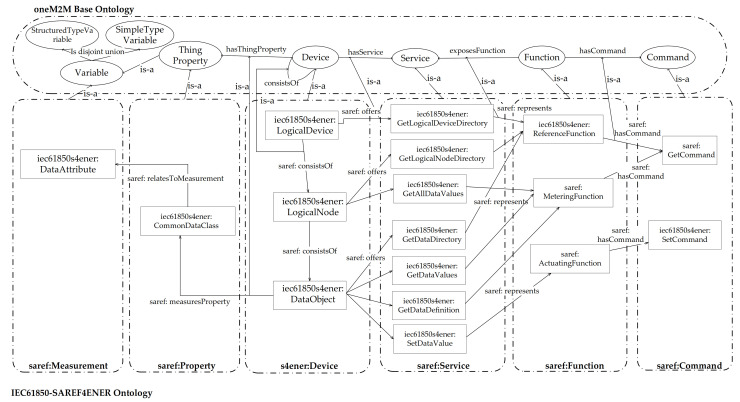
Common Ontology between IEC61850-SARF4ENER and oneM2M Base Ontology.

Figure 12 shows the common ontology herein presented.

In the upper section, the classes of the oneM2M base ontology have been grouped. In the lower section, the classes belonging to the IEC61850-SAREF4ENER ontology have been grouped according to the relevant semantic, given by the saref classes they inherited from.

To establish the semantic relationship between the two ontologies, the semantic “is-a” relation was used, as defined for the oneM2M Base ontology [49] and already explained in the Section 5.1.

Let’s start the description of the mapping, from the iec61850s4ener classes inherited from s4ener: Device class. As shown by Figure 12, the s4ener:Device group of classes includes iec61850s4ener:LogicalDevice, iec61850s4ener:LogicalNode and iec61850s4ener: DataObject. All these classes have been semantically related to the oneM2M device class; this is shown by the “is-a” relation between the s4ener:Device group and the oneM2M Device class. The saref:consistsOf present in this group has been related to the oneM2M consistsOf, as done in [42].

In the saref:Property group, the only class present is iec61850s4ener:CommonDataClass, which has been related to oneM2M Thing Property.

Considering the saref:Measurement group, it has been assumed to relate the iec61850s4ener:DataAttribute to the oneM2M Variable class, as shown by Figure 12.

The saref:Service group includes the classes defined in IEC61850-SAREF4ENER to represent the services offered by LogicalDevice, LogicalNode and DataObject; all these classes have been related to the oneM2M Service class, as shown by Figure 12.

In the saref:Function group, the iec61850s4ener:ReferenceFunction, iec61850s4ener:MeteringFunction and iec61850s4ener:ActuatingFunction, relate to the oneM2M Function class.

The iec61850s4ener:SetCommand has been mapped to oneM2M Command class, as done in [42] for the saref:GetCommand class.

## 7. Interworking Proposal

This paper presents a semantic interoperability proposal between oneM2M and IEC 61850, based on the definition of an interworking scheme from IEC 61850 to oneM2M, with exposure of IEC 61850 information model into the oneM2M domain. The interworking solution has been based on the oneM2M ontology-based interworking described before; on account of this choice, it foresees the use of the oneM2M IPE and the common ontology here proposed and presented in Section 6.

**Figure 13:**
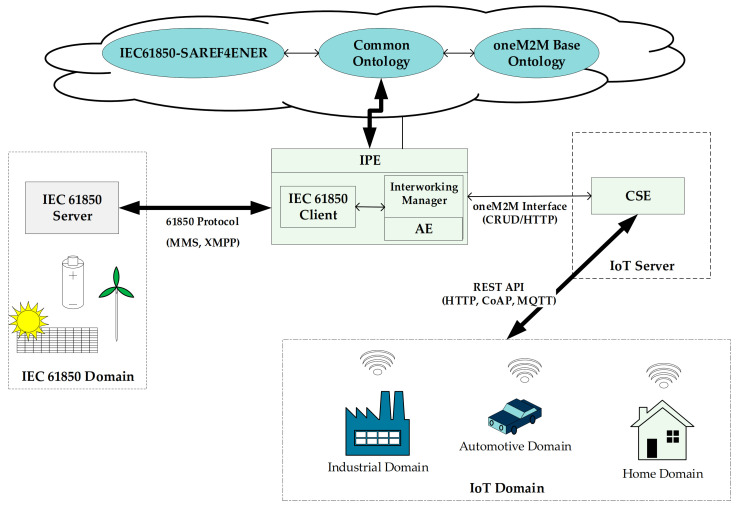
Interworking Architecture based on IPE and Common Ontology.

The architecture depicted by Figure 13 highlights the main elements involved in the proposal. On the left, an IEC 61850 Server refers to IEC 61850 domain consisting for example of a wind farm, a hydroelectric power plant or a distributed power source. IEC 61850 Server contains data instances of the relevant domain (e.g., LD, LN, DO and the relevant services).

IPE allows interworking between IEC 61850 and oneM2M-based IoT domains. Figure 13 shows the architectural choices made for the IPE. Interworking manager is the core of the IPE, managing the entire set of interworking activities here proposed. IPE features the presence of an IEC 61850 client able to access data and services of the IEC 61850 Server through interfaces over a TCP-IP network (e.g., MMS, XMPP). The last element foreseen for the IPE, is an application entity (AE) that provides the communication interface with a registrar CSE, i.e., a CSE where this AE has been registered. It has been assumed that this CSE contains the oneM2M resources mapping the information maintained by the IEC 61850 Server, according to the oneM2M ontology-based interworking and the common ontology proposed in this paper between IEC61850-SAREF4ENER and oneM2M base ontology. Communications between the AE present inside the IPE and registrar CSE is based on oneM2M interface; in this paper it has been assumed to realize this interface only using CRUD operations realized by HTTP methods as foreseen by the oneM2M standard.

As shown by Figure 13, it has been assumed that the IoT devices and applications in the IoT domains access an IoT Server made up by the CSE maintaining the oneM2M resource mapping information of the IEC 61850 Server. This allows the access to information and services of the IEC 61850 domain, as it will be explained in the remainder of this section.

In the following, a detailed description of the proposal will be given in order to allow the reader to a better understanding. Description will be split into subsections; the first one will highlight the basic assumptions made for the proposal about the realization of the oneM2M ontology-based Interworking. The second subsection will be aimed to describe the activities foreseen for the IPE at start-up, i.e., before the IPE could actually perform its interworking role. The last subsection will deal with the run-time activities foreseen for the IPE in order to allow every device in the oneM2M domains to access information and services offered by the IEC 61850 domain.

### 7.1. Realisation of the oneM2M Ontology-Based Interworking

The main assumption of the paper is that to adopt the oneM2M ontology-based interworking described in Section 5.2. This choice implies that a common ontology between ontologies of IEC 61850 and oneM2M must exist; the paper has presented a proposal of common ontology between the ad-hoc defined IEC61850-SAREF4ENER ontology and the oneM2M base ontology, but the interworking activities proposed in this paper may be applied also for other common ontologies which may be defined in the future. Proposing here other ontologies for the IEC 61850 could be possible, but it was avoided by the author as it was not the main goal of the paper. Using another ontology for the IEC 61850 do not involve substantial modification to the interoperable and interworking solution here presented.

**Figure 14:**
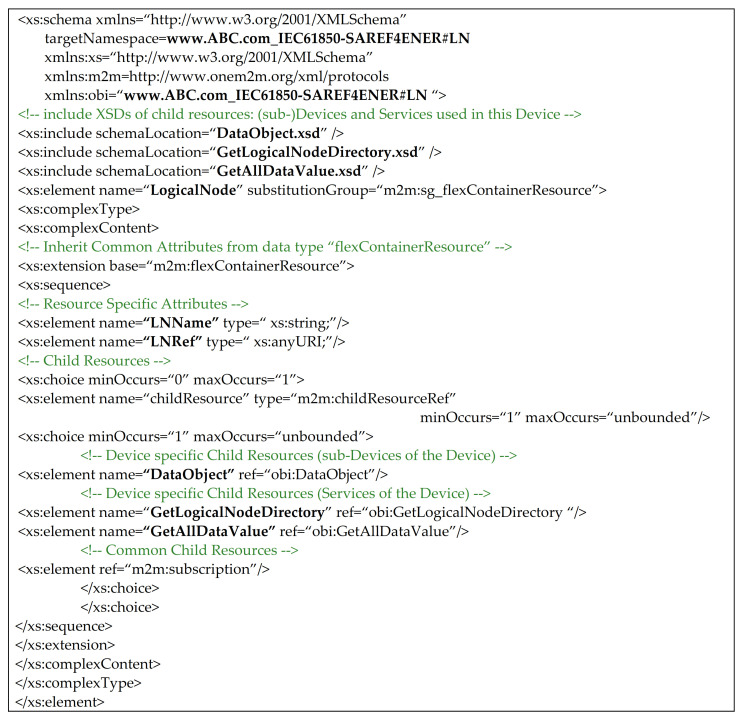
XSD file representing the iec61850s4ener: LogicalNode class as a <flexContainer> oneM2M resource.

Another assumption is that, according to [54], XSD files for each kind of oneM2M resource used in the common ontology to map IEC 61850 resources must be available. For this reason, a set of schemas has been defined for each class defined inside the IEC61850-SAREF4ENER ontology. Just as an example, the XSD file representing the iec61850s4ener: LogicalNode class is shown in Figure 14. Considering the IEC61850-SAREF4ENER ontology, the class iec61850s4ener:LogicalNode represents the IEC 61850 LN; in the common ontology this class corresponds to a device class in the oneM2M ontology (see Figure 12). According to [54], a device class may be represented in oneM2M by a <flexContainer>, as pointed out by the legenda of Figure 7. This means that XSD schemas relevant to the class iec61850s4ener:LogicalNode has been built assuming to use a <flexContainer> oneM2M resource for the relevant representation in the oneM2M domain. As said in Section 5.2 and as shown by Figure 14, the XSD schema is made up by several subsections. The first one contains the list of the XSD files describing the child oneM2M resources. On the basis of the common ontology given by Figure 12, it is clear that iec61850s4ener:LogicalNode is related to the classes iec61850s4ener:GetLogicalNodeDirectory, iec61850s4ener:GetAllDataValues and iec61850s4ener:DataObject. On the basis of what said, the first section of XSD schema in Figure 14 must contains the list of the XSD files relevant to these classes. The second section of XSD schema shown in Figure 14, exactly define the <flexContainer> structure that must be used to represent the iec61850s4ener:LogicalNode class into the oneM2M CSE. First of all, this section describes the non-common attributes hold by this resource; they are LNName (defined as xs:string) and LNRef (defined as xs:anyURI), according to the IEC61850-SAREF4ENER ontology (Figure 12). Then a particular subsection named “Child Resource” is present; it is aimed to define the schema of the <flexContainer>. In particular it defines that this <flexContainer> features a sub-Device relevant to the iec61850s4ener:DataObject, and it features two Services relevant to iec61850s4ener:GetLogicalNodeDirectory and iec61850s4ener:GetAllDataValue. Finally, child resources common to oneM2M are specified in the last part of this subsection; among them there is the <subscription> resource.

### 7.2. IPE Activities at Start-Ip

The first activity that the IPE must perform on the CSE available to represent the IEC 61850 resources, is that to register its AE; the result is the creation of a oneM2M <AE> child resource of the <CSEBase> resource representing the Registrar CSE.

After registration, a <container> resource must be created in the registrar CSE in order to realize the XSD storage described in Section 5.2. This <container> will be populated by the IPE with <contentInstance> child-resources relevant to the XSD files representing the mapping between iec61850-saref4ener classes into oneM2M resources, as explained in Section 5.2. The previous subsection pointed out that the set of these files for the mapping here proposed are available.

**Figure 15:**
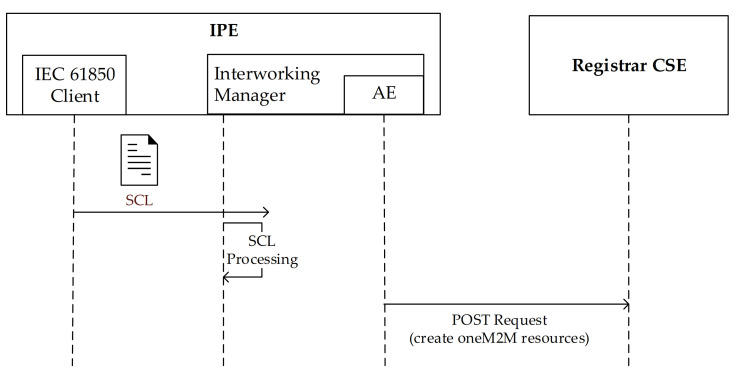
Operations performed by IPE at start-up.

The next step, perhaps the most important one, to be realized by the IPE is the population of the oneM2M resources representing the actual IEC 61850 resources maintained by the server. According to IEC 61850 standard, each client maintains information about the actual structure of the information model of the IEC 61850 server (e.g., the set of LDs, LNs, data objects and services) using SCL format. It is required that the IEC 61850 Client inside the IPE, passes this information to the interworking manager. Interworking manager is in charge to process the SCL file received by the client. For each IEC 61850 resource found in the SCL file, a specialization of <flexContainer> resource will be instantiated in the Registrar CSE as child resource of <AE> representing the IPE, in accordance with each XSD file found in the XSD repository. Figure 15 shows what said until now and it points out that the exchange of SCL-based information between IEC 61850 client and interworking manager inside IPE; furthermore, it highlights the HTTP POST requests sent to the registrar CSE in order to create oneM2M resources.

**Figure 16:**
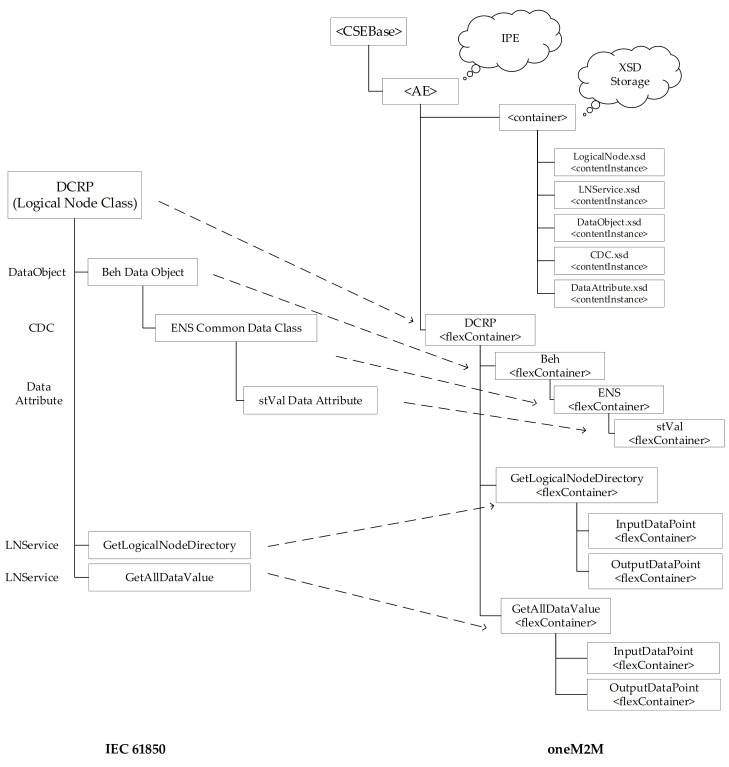
Example of the oneM2M resources created by IPE in the Registrar CSE at Start-up.

Figure 16 shows an example of the final result achieved by the specialization of <flexContainer> resources performed by the IPE, according to the information flow shown by Figure 15. As it is possible to see, Figure 16 shows the <AE> resource created after the registration of the IPE, the <container> resource used to realize the XSD storage group, and the <contentInstance> resources relevant to the XSD schemas representing the IEC61850-SAREF4ENER classes (e.g., LogicalNode.xsd, LNService.xsd, and DataObject.xsd).

In this example, it has been assumed to consider a real IEC 61850 scenario featured by a DER. In IEC 61850 7-420 [38], the DER plant electrical connection point (ECP) logical device defines the characteristics of the DER plant at the point of electrical connection between one or more DER units and any electric power system (EPS), including isolated loads, microgrids, and the utility power system. ECP logical device would include several logical nodes, among which the DER plant corporate characteristics at each ECP (DCRP), including ownership, operating authority, contractual obligations and permissions, location, and identities of all DER devices connected directly or indirectly at the ECP. Due to this assumption, Figure 16 on the left shows the structure of the DCRP logical node; details about the elements contained in this structure may be achieved in IEC 61850 7-420 [38]. On the right, Figure 16 points out the CSE resource tree after DCRP logical node instances creation. Due to lack of space only main instances are considered and shown.

In order to represent the DCRP logical node a specialization of <flexContainer> is used, according to the XSD schema shown by Figure 14; Figure 15 shows the relationship between IEC 61850 DCRP LogicalNode and the DCRP <flexContainer> resource instanced by the IPE to expose it in oneM2M environment.

Beh is a DataObject belonging to the DCRP LN; according to the DataObject.xsd (not shown in this paper for space reason), another specialization of <flexContainer> is used to represent it and its attributes.

Enumerated status (ENS) is a common data class used to describe Beh DataObject through a group of data attribute (stVal). According to CDC.xsd and DataAttribute.xsd schemas, the ENS common data class and stVal data attribute are represented in the CSE by the ENS and StVal specializations of <flexContainer> resource, as shown by Figure 16.

IEC 61850 Services of DCRP are represented in oneM2M by two specialization instances of <flexContainer> resource as child resource of DCRP <flexContainer>. InputDataPoint and OutputDataPoint <flexContainer> are needed for the services invocation, as described in next section.

### 7.3. IPE Activities at Run-Time

Once the start-up has been completed and the registrar CSE has been populated with the oneM2M resources representing the actual IEC 61850 information model, the IPE may actually offer its interworking services to the IoT devices and applications in the oneM2M domain.

According to the guidelines given by oneM2M ontology-based Interworking [54] about the data exchanges performed by the IPE between two different domains, several interworking activities have been defined for the IPE in this proposal. In the following, details about the main activities foresees for the IPE will be given, pointing out the data flows between the several entities of the interworking architecture shown by Figure 13.

#### 7.3.1. Retrieving Information Produced by IEC 61850 Server

Each time an information changes in the IEC 61850 server (e.g., the value of a certain attributes is updated), this change must be detected by the IPE and the registrar CSE must be updated accordingly; in this way each change in the IEC 61850 can be made available to whatever IoT device and application compliant with oneM2M. This is realized adopting the subscription mechanism defined by oneM2M and explained in Section 5.

**Figure 17:**
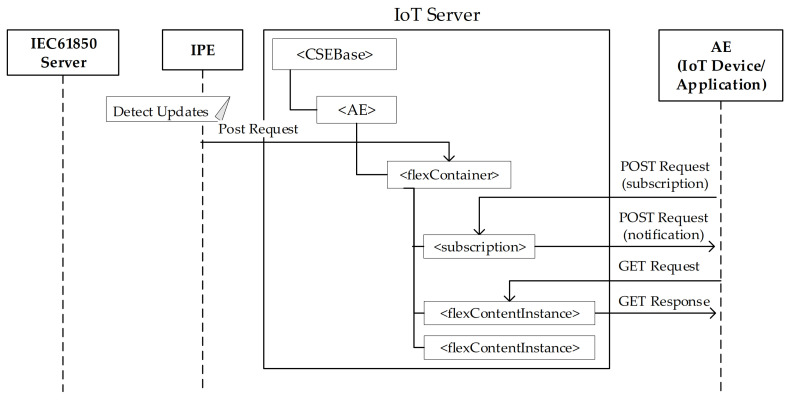
Retrieving information produced by IEC 61850 Server.

Figure 17 shows on the right side an AE which may belong to an IoT oneM2M-based device of the field domain or it may belong to an application in the IoT infrastructure domain (see Figure 3). The IoT server is made up by a CSE, as said before. The <CSEBase> resource represents the CSE and the <AE> child resource is due to the IPE registration made at the start-up phase, as described in the previous section.

For each IEC 61850 resource maintained by the IEC 61850 server and mapped into the registrar CSE, the IPE is in charge to detect updates of value attributes. For each update, the IPE will send a post request in order to publish this update in the <flexContainer> resource representing the IEC 61850 resource which has been updated; Figure 17 shows this <flexContainer> resource, inside the IoT server. According to the oneM2M standard [45], the publication of each new information for a <flexContainer> is realized through the creation of a new <flexContentInstance> instance inside the <flexContainer> resource. Figure 17 shows several <flexContentInstance> resources created to publish the new values of the IEC 61850 resource each time it is updated.

According to [45], the AE on the right side of Figure 17 is able to get the updated values, according to the following mechanisms. First of all, the AE in the oneM2M domain subscribes itself to the <flexContainer> resource from which it is interested to received information. This is done through the Create <subscription> request service sent to the CSE through a POST request, as shown by the Figure 17.

On the basis of this subscription, the CSE will notify its subscriber (i.e., the AE on the left of Figure 17) of any events under the subscribed resource (e.g., when a <flexContentInstance> resource is created under the <flexContainer>), through a POST Request containing the notification of the update, as shown by the Figure 17. On the receipt of this notification, the AE sends a HTTP GET Request to the IoT Server with the URL linked to the <flexContentInstance> resource it wants to get. The IoT Server sends back the HTTP GET Response with the updated value contained by the <flexContentInstance>.

#### 7.3.2. Updating Information to IEC 61850 Server

A device or an application in the oneM2M domain may have the need to change the value of one or more information maintained in the CSE and relevant to IEC 61850 resources. In this case, it is requested that the IPE should extend these updates to the IEC 61850 server.

**Figure 18:**
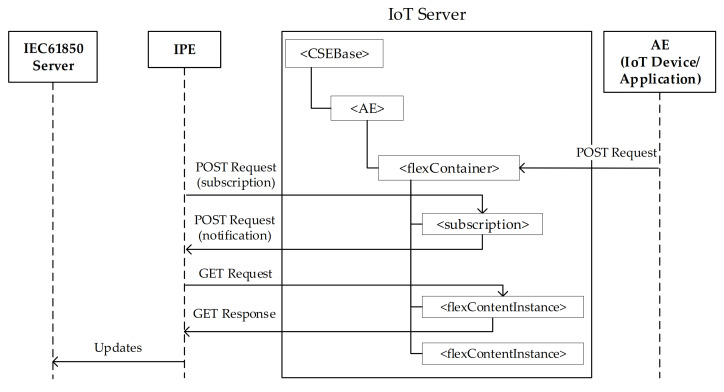
Updating information to IEC 61850 Server.

Figure 18 illustrates the data exchange defined in the proposal in order to reach this aim. As done for the Figure 17, on the left side the AE residing in the IoT oneM2M device of the field domain or in the application of the oneM2M infrastructure domain is shown. Furthermore, the Figure 18 shows a <flexContainer> resource representing an IEC 61850 resource. Each time the AE would like to update this resource, an HTTP POST Request allows to create a new <flexContentInstance> under the <flexContainer>, containing the update introduced.

In order each update made inside the IoT domain is extended to the IEC 61850 Server, it is required that the IPE creates a <subscription> resource under the <flexContainer> resource, in order to get alerts as soon as new <flexContentInstance> resource is created as child. For each notification received by the IPE, it can get the update through the GET request/response exchange, as described in the previous sub section. The information retrieved from the CSE, will be send by the IPE to the IEC 61850 Server in order to apply this update in the IEC 61850 domain. In this case the IPE uses the IEC 61850 Client inside it, off course.

#### 7.3.3. Invoking Services Running on IEC 61850 Server from oneM2M Domain

The proposal here present allows a oneM2M device/application to invoke a service on a IEC 61850 server, specifying the input data associated to the service invoked (e.g., a command). It is possible that the IEC 61850 server reacts to the invoked service with a response (e.g., the status result of the service), sending back this response after having completed the invoked service.

The mechanism adopted to realize the service invocation just described is based on what stated in document [54]. It is important to recall that in the proposal here presented, a oneM2M Service mapping IEC 61850 service is realized by a <flexContainer> resource, having two <flexContainer> child resources representing InputDataPoint and OutputDataPoint. According to [54], each time a device/application in the oneM2M domain would like to invoke a service, it must issue a POST request on the InputDataPoint updating the relevant value; the results of the service invocation (if foreseen for the service) will be given by the new value of the OutputDataPoint.

**Figure 19:**
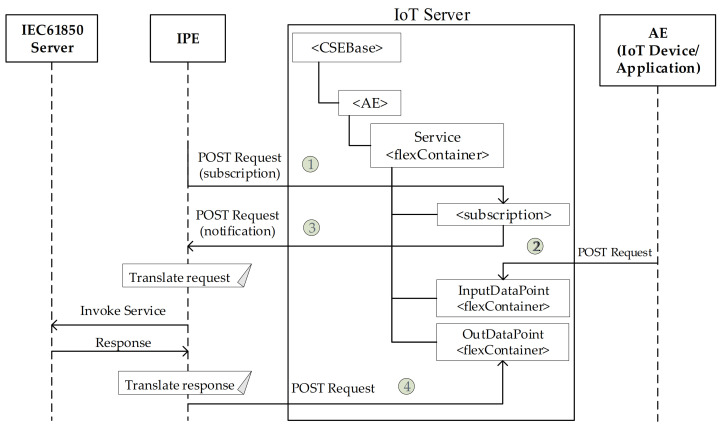
Invoking services running on IEC 61850 Services from oneM2M domain.

Figure 19 shows the IoT Server and the relevant CSE, where a <flexContainer> resource represents an IEC 61850 service; the <flexContainer> resources realizing the oneM2M InputDataPoint and OutputDataPoint are also present. It points out the communication flow required for the service invocation. It is assumed that IPE must be subscribed to the <flexContainer> representing the service to be invoked; as said before, this is done by a POST Request containing a Subscription request (see point number 1 in Figure 19).

When the device/application in the oneM2M domain wants to invoke a service on the IEC 61850 server (e.g., an actuation command), the relevant AE updates the <flexContainer> that represents the service with the new value for the InputDataPoint; this is done by the POST request shown in the right part of Figure 19 (point number 2). The CSE subsequently notifies the IPE about the changed value for InputDataPoint; this happens on account of the subscription previously created, and it is realized through a notification sent by a POST request (point number 3 in Figure 19).

The IPE invokes the command at the IEC 61850 server (through the internal client), sending the data of the InputDataPoint.

Depending on the kind of service invoked, the IEC 61850 server may send back a response (e.g., a reporting) relevant to the previous invoked service. When this happens, the IPE receives the response sent by the IEC 61850 Server, and then it updates the corresponding <flexContainer> that represents the Service with the new value for the OutputDataPoint (point 4). The CSE may subsequently notify the AE about the changed value for OutputDataPoint, if the AE was subscribed to the relevant <flexContainer>; this last information flow is not showed in Figure 19.

## 8. Final Remarks

This paper had two different aims. First of all, an interworking scheme between IEC 61850 and oneM2M has been proposed. A solution to introduce semantic interoperability in this interworking scheme has been also given. The proposal is mainly based on the oneM2M Ontology-based Interworking and on the concept of common ontology between different ontologies. The proposal has required the definition of ad-hoc ontology for the IEC 61850 system, called IEC61850-SAREF4ENER; furthermore, the definition of a common ontology between IEC61850-SAREF4ENER and oneM2M base ontology has been proposed. The paper has described the main features of the solution proposed in terms of data exchanges that can be realized between IEC 61850 and oneM2M domains. As explained in the paper, the main actor in the proposal is the standard oneM2M IPE. The paper has presented a detailed description of the internal architecture here proposed, made by several entities.

The proposal here presented has several advantages and practical implications. First of all, it represents a first step towards enabling IoT technologies in the smart grid domain; the interworking solution here presented is also interoperable and this allows to cover a gap in the current literature, due the lack of such kind of solutions in the smart grid. As said, the proposal is based on the use of the standard oneM2M IPE, which was specialised to realize a proxy between oneM2M-based IoT and IEC 61850 domains. From this point of view the paper has a strong impact on the oneM2M standard definition, as the results presented may be used to enrich the current standard oneM2M documents. At this moment, description of the oneM2M ontology-based interworking in the oneM2M standard documents presents a very limited number of IPE specialization, and the Smart Grid specialization proposed in the paper could be of interest if added as another example of specialization in a real domain.

It is important to point out, that the proposal does not introduce an overhead in the interworking operations of the IPE. The proposal is based on the same procedures defined by the oneM2M standard for the IPE, although they were specialized for the specific interworking scenario related to the mapping between IEC 61850 and oneM2M.

In order to validate the proposed architecture a prototype has been realized. It is based on open source libraries. In particular, the “iec-61850bean” [56] has been used to realize both the IEC 61850 client and server; implementation of the AE inside the interworking manager has been based on “OpenMTC SDK” [57].

## Figures and Tables

**Table 1:** Classes of IEC 61850 Information Model.

GenClass	Attribute Name	Services
GenLogicalDevice	LDName, LogicalNode [1…n]	GetLogicalDeviceDirectory
GenLogicalNode	LNName, LNRef, DataObject [1…n]	GetLogicalNodeDirectory, GetAllDataValues
GenDataObject	DataObjectName, DataObjectRef, m/o/c, DataObjectType	GetDataValues, SetDataValues, GetDataDirectory, GetDataDefinition
GenCommonData	CDC-ID, DataAttribute [0…n]	
GenDataAttribute	DataAttributeName, FC, m/o/c	

## Data Availability

The study did not report any data.
